# Repeated measurements of cerebral blood flow in the left superior temporal gyrus reveal tonic hyperactivity in patients with auditory verbal hallucinations: a possible trait marker

**DOI:** 10.3389/fnhum.2013.00304

**Published:** 2013-06-25

**Authors:** Philipp Homan, Jochen Kindler, Martinus Hauf, Sebastian Walther, Daniela Hubl, Thomas Dierks

**Affiliations:** ^1^Department of Psychiatric Neurophysiology, University Hospital of Psychiatry, University of BernBern, Switzerland; ^2^Support Center for Advanced Neuroimaging, Institute of Diagnostic and Interventional Neuroradiology, Inselspital, University of BernBern, Switzerland

**Keywords:** schizophrenia, auditory verbal hallucinations, cerebral blood flow, arterial spin labeling, superior temporal gyrus, longitudinal study

## Abstract

**Background:** The left superior temporal gyrus (STG) has been suggested to play a key role in auditory verbal hallucinations (AVH) in patients with schizophrenia.

**Methods:** Eleven medicated subjects with schizophrenia and medication-resistant AVH and 19 healthy controls underwent perfusion magnetic resonance (MR) imaging with arterial spin labeling (ASL). Three additional repeated measurements were conducted in the patients. Patients underwent a treatment with transcranial magnetic stimulation (TMS) between the first 2 measurements. The main outcome measure was the pooled cerebral blood flow (CBF), which consisted of the regional CBF measurement in the left STG and the global CBF measurement in the whole brain.

**Results:** Regional CBF in the left STG in patients was significantly higher compared to controls (*p* < 0.0001) and to the global CBF in patients (*p* < 0.004) at baseline. Regional CBF in the left STG remained significantly increased compared to the global CBF in patients across time (*p* < 0.0007), and it remained increased in patients after TMS compared to the baseline CBF in controls (*p* < 0.0001). After TMS, PANSS (*p* = 0.003) and PSYRATS (*p* = 0.01) scores decreased significantly in patients.

**Conclusions:** This study demonstrated tonically increased regional CBF in the left STG in patients with schizophrenia and auditory hallucinations despite a decrease in symptoms after TMS. These findings were consistent with what has previously been termed a trait marker of AVH in schizophrenia.

## Introduction

In schizophrenia, auditory verbal hallucinations (AVH) comprise a critical domain. The 1-month prevalence of these hallucinations exceeds 70% (Sartorius et al., 1986), and, in 25–30% of patients, these perceptions are resistant to medication, resulting in functional disability and a low quality of life (Shergill et al., 1998; Copolov et al., 2004). The development of new therapeutic strategies (Homan et al., 2011) is urgent and would benefit from a better understanding of the neurophysiology of AVH.

The results of resting perfusion and functional imaging studies have implied that AVH are associated with altered neuronal activity in cerebral areas that are responsible for language production and perception (Allen et al., 2008; Strik and Dierks, 2008). AVH have been shown to be positively correlated with resting-state perfusion (regional cerebral blood flow, CBF) in the medial temporal lobe (Liddle et al., 1992), the superior temporal lobe (Gur et al., 1995), and the anterior cingulate cortex (Lahti et al., 2006) and negatively correlated with perfusion in the hippocampus/parahippocampus (Lahti et al., 2006). In addition, when CBF has been measured before and after interventions with transcranial magnetic stimulation (TMS), it has been found to be decreased at the stimulation site, which is the left superior temporal gyrus (STG), and in interconnected regions and increased in the contralateral cortex and the frontal lobes after 10 days of TMS treatment (Horacek et al., 2007). In a previous study, we found an association of favorable TMS treatment effects and decreased neuronal activity in the primary auditory cortex, Broca's area, and the cingulate gyrus (Kindler et al., 2013), suggesting that CBF might be a biological marker for the effectiveness of TMS. Furthermore, the CBF in the left STG before treatment predicted the response to TMS, indicating that resting perfusion measurements before treatment might be appropriate for differentiating possible responders and non-responders to TMS (Homan et al., 2012). However, those CBF measurements were limited to only one time point, which was before treatment, and the time courses of the CBF and the psychopathological symptoms were not assessed with repeated measurements. Several studies have investigated the clinical severity of hallucinations longitudinally (Arndt et al., 1995; Marengo et al., 2000; Mancevski et al., 2007; Chang et al., 2009; Schneider et al., 2011). Until now, it has been unclear in which way the neuronal activity followed the clinical course of AVH longitudinally in individual patients. Regions with neuronal activity that follow the clinical course may be regarded as state-dependent, whereas areas that demonstrate continuous aberrant activity compared to those in non-hallucinators and healthy subjects may be regarded as trait-specific.

**Figure 1:**
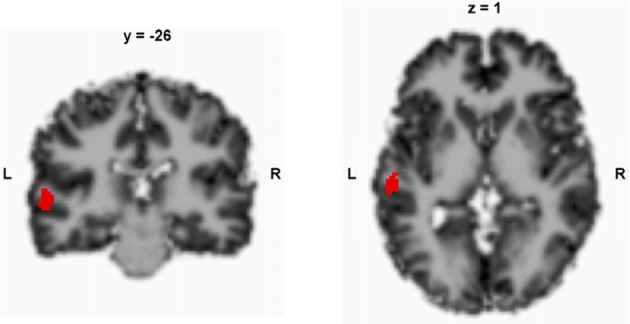
**Predefined region of interest in the left superior temporal gyrus**. The local maximum peak values were retrieved from a previous study (Homan et al., 2012); according to SPM 8, *x, y, z* = −58, −26, 4; *x, y, z* coordinates in Talairach space.

In this study, we repeatedly measured CBF in a region of interest (ROI) that has been previously identified (left STG, Figure 1) to exhibit predictive CBF before TMS treatment in patients with medication-resistant AVH (Homan et al., 2012). Patients were treated with TMS according to a 10-day-treatment protocol between the first and the second measurement (Kindler et al., 2013). Our aim was to gain insights into the fluctuations of CBF and symptoms. In order to measure CBF, we used magnetic resonance (MR) arterial spin labeling (ASL), which is a MR technique that provides a direct quantitative measure of CBF (Horn et al., 2009; Jann et al., 2010; Viviani et al., 2010; Walther et al., 2012). ASL is a non-invasive technique that has been shown to provide converging results with those that have been obtained by invasive positron emission tomography perfusion imaging (Xu et al., 2010). It thus can more easily and less invasively be applied in situations that require repeated examinations.

In this study, we were interested in whether the proposed responsiveness of regional CBF in the left STG to TMS (Homan et al., 2012) was a stable (trait-like) feature over time or whether there were fluctuations in neuronal activity across time. Additionally, we compared the initial measurements of the series with measurements in 19 healthy controls. Based on the results of a recent meta-analysis that have suggested that the neuronal activity in the left STG might be a trait-like feature in AVH (Kuhn and Gallinat, 2012), we hypothesized that the CBF in the left STG would be increased compared to healthy controls and would not change across time.

## Methods

### Patients and clinical investigation

The same patient population as that described in Homan et al. (2012) was used. However, only 11 patients were willing to undergo repeated measurements after TMS treatment. The patients who were included in the study had a diagnosis of schizophrenia or schizoaffective disorder according to the International Statistical Classification of Diseases and Related Health Problems (ICD)-10, medication-resistant AVH, and ages of 18–65 years, and they were right-handed. The right-handed healthy control subjects had no history of any psychiatric disorders and no major psychiatric conditions in their first-degree relatives (*n* = 19). The exclusion criteria were MR contraindications and any medical disorders other than schizophrenia or schizoaffective disorder in the patients. None of the subjects reported substance misuse in the 4 weeks before or during the study. All patients underwent identical diagnostic procedures, including the MR protocol. The diagnostic procedures were conducted based on the clinical interviews and psychiatric histories. The psychopathology assessment, which consisted of the Positive And Negative Syndrome Scale (PANSS) (Kay et al., 1988) and the Psychotic Symptom Rating Scale (PSYRATS) (Haddock et al., 1999), was performed on the same occasion as the ASL. In order to ensure that all subjects were right-handed, the Edinburgh Handedness Scale (Oldfield, 1971) was assessed. The investigation was conducted in accordance with the Declaration of Helsinki and was approved by the local ethics committee. All subjects provided informed written consents to participate in the study.

### Study procedure

Patients and controls were measured with ASL at baseline. The psychopathology in the patients was assessed. Patients then underwent 10 days of TMS treatment. Within 36 h after the TMS treatment, the patients' psychopathology and CBF with ASL were assessed again. Patients underwent two follow-up examinations of perfusion MRI and psychopathology 4 weeks and 8 weeks post-TMS.

### TMS protocol

The TMS protocol has been described elsewhere (Homan et al., 2012; Kindler et al., 2013). Briefly, the target region for TMS was the sensorimotor speech region, which is called the Sylvian parietotemporal (Spt) area and which is located in the Sylvian fissure at the parietotemporal boundary. We applied a modified version of the language processing task that was developed by Hickok et al. (2009) to localize the Spt area for TMS treatment in a functional MR imaging (fMRI) scan of each patient in the first MR measurement. This fMRI measurement was conducted immediately after the ASL measurement. The individual's fMRI activation map was superimposed on reconstructed anatomical mesh, and the target point of the Spt area was marked for TMS. We used a custom TMS stimulator (MagPro R 100, Medtronic Functional Diagnostics A/S, Skovlunde, Denmark) to generate repetitive biphasic magnetic pulses with a figure-8 coil (Magnetic Coil Transducer MC-B70, Medtronic Functional Diagnostics A/S). The individual resting motor threshold was identified by stimulation of the motor cortex with single TMS pulses until a movement of the contralateral thumb was detected (Schutter and Van Honk, 2006). TMS pulse intensity was then adjusted to 90% of the resting motor threshold. Patients were randomly assigned to receive 1 Hz (*n* = 7) or theta burst (*n* = 4) TMS. The target area was stimulated for 10 consecutive days in both groups. Common TMS safety protocols were applied according to international safety standards (Rossi et al., 2009). A frameless, ultrasound-based, stereotactic system was used for neuronavigation (Brainvoyager™ TMS Neuronavigator System, Brain Innovation B.V., Maastricht, Netherlands) (Sack et al., 2009).

### MRI data analysis: ASL

MRI was conducted on a 3.0-Tesla whole-body MRI system (Magnetom Trio, Siemens Medical Systems, Erlangen, Germany) with a standard 12-channel radiofrequency head coil. High-resolution three-dimensional (3D) structural MRI and ASL were acquired in each session. T1-weighted 3D magnetization prepared-rapid gradient echo (MP-RAGE) scans were recorded (number of slices, 176; matrix, 256 × 256; slice thickness, 1 mm; voxel size, 1 × 1 × 1 mm^3^), and they served as high-resolution 3D anatomical templates for coregistration with the functional data. A pseudocontinuous ASL (pCASL) technique was used to measure CBF (Wang et al., 2005). In this gradient-echo echo-planar imaging sequence, interleaved images with and without labeling were acquired. A delay of 1250 ms was applied between the end of the labeling pulse (label time, 1600 ms) and image acquisition (slice acquisition time, 45 ms) in order to reduce transit artifact (field of view, 220 mm^2^; matrix, 64 × 64; repetition time/echo time, 4000/18 ms; flip angle, 90°; and labeling efficiency a, 0.95). A total of 14 slices (voxel size, 3.4 × 3.4 × 6 mm^3^; slice gap, 1.5 mm) was acquired in the anterior and posterior commissure line from inferior to superior in sequential order. The pCASL scan comprised 80 acquisitions. The ASL data analysis was performed in a manner that was similar to that described by Homan et al. (2012). Briefly, we used aslm (Homan et al., 2012) (downloadable at http://aslm.sourceforge.net), which is based on MATLAB® (MATLAB version 8, release 14; The MathWorks, Inc., Natick, MA, USA) and statistical parametric mapping (SPM 8, Wellcome Department of Imaging Neuroscience, London, England; www.fil.ion.ucl.ac.uk/spm8). All ASL time series were first realigned to correct for motion artifacts. We calculated a flow-time series by subtracting the labeling images from the control images and subsequently computed mean CBF images for each subject (Federspiel et al., 2006). Each individual subjects' T1 anatomy was segmented into gray matter (GM) and white matter (WM). The mean ASL images were then coregistered to the GM-segmented T1 images. T1, GM, WM, and ASL images were normalized to the SPM MNI T1 template. ASL images were spatially smoothed with a 3D 8-mm full-width at half-maximum Gaussian kernel. Data were *z*-transformed [*z* = (voxel CBF—global GM CBF)/SD] in order to remove sources of variance that were caused by differences in the global mean CBF between acquisitions and corrected for GM by using GM segments as inclusive masks.

### Statistical analysis: CBF

A global and assumption-free investigation of the whole-brain CBF was computed. CBF values were then extracted from the a priori-defined ROI in the left STG that corresponded to the finding described in Homan et al. (2012) with aslm. Therefore, the ROI was used as an inclusive mask of each subject's ASL measurement. The mean regional blood flow of the ROI was calculated by taking the mean of all voxels inside the mask. A full-factorial linear mixed model with a restricted maximum likelihood estimation was then computed with the pooled global and regional CBF values of the baseline measurements as outcome measures and diagnosis, localization (global, regional), and the diagnosis-by-localization interaction as fixed effects. The longitudinal patient data were then examined separately in full-factorial mixed models with restricted maximum likelihood estimation in order to examine the effects of localization, time, and medication (chlorpromazine dose equivalent, CPZE) on the CBF outcome measures. In order to additionally assess the effect of psychopathology on CBF, PSYRATS, and PANSS were included as additional fixed effects in these models. For the outcome measures of psychopathology (PSYRATS, PANSS), the effects of time, TMS protocol, and medication were assessed for the first 2 observations (pre- and post-TMS treatment). For the additional observations (at *t* = 2, 3, and 4), the effects of time and medication were assessed on the outcome measures of the PSYRATS and PANSS. In order to account for the different intervals between the measurements after TMS treatment, interval was also included as a fixed effect in these longitudinal models. Furthermore, these models included a subject effect in order to account for the repeated measurements. The Schwarz Bayesian criteria were used to determine the best fitting covariance structure for each set of measures in cases where the typical compound symmetry approach that was used by ANOVA did not provide the optimal structure for the extant data. A heterogeneous variance first-order autoregressive covariance structure proved to be appropriate for all mixed models. The *post-hoc t*-tests involved a Tukey correction for multiple comparisons. SAS 9.2 (SAS Institute, Inc., Cary, NC, USA) was used for all analyses. The means are reported with their associated standard deviations (SDs). Statistical significance was set at *p* < 0.05.

## Results

### Clinical data

**Figure 2:**
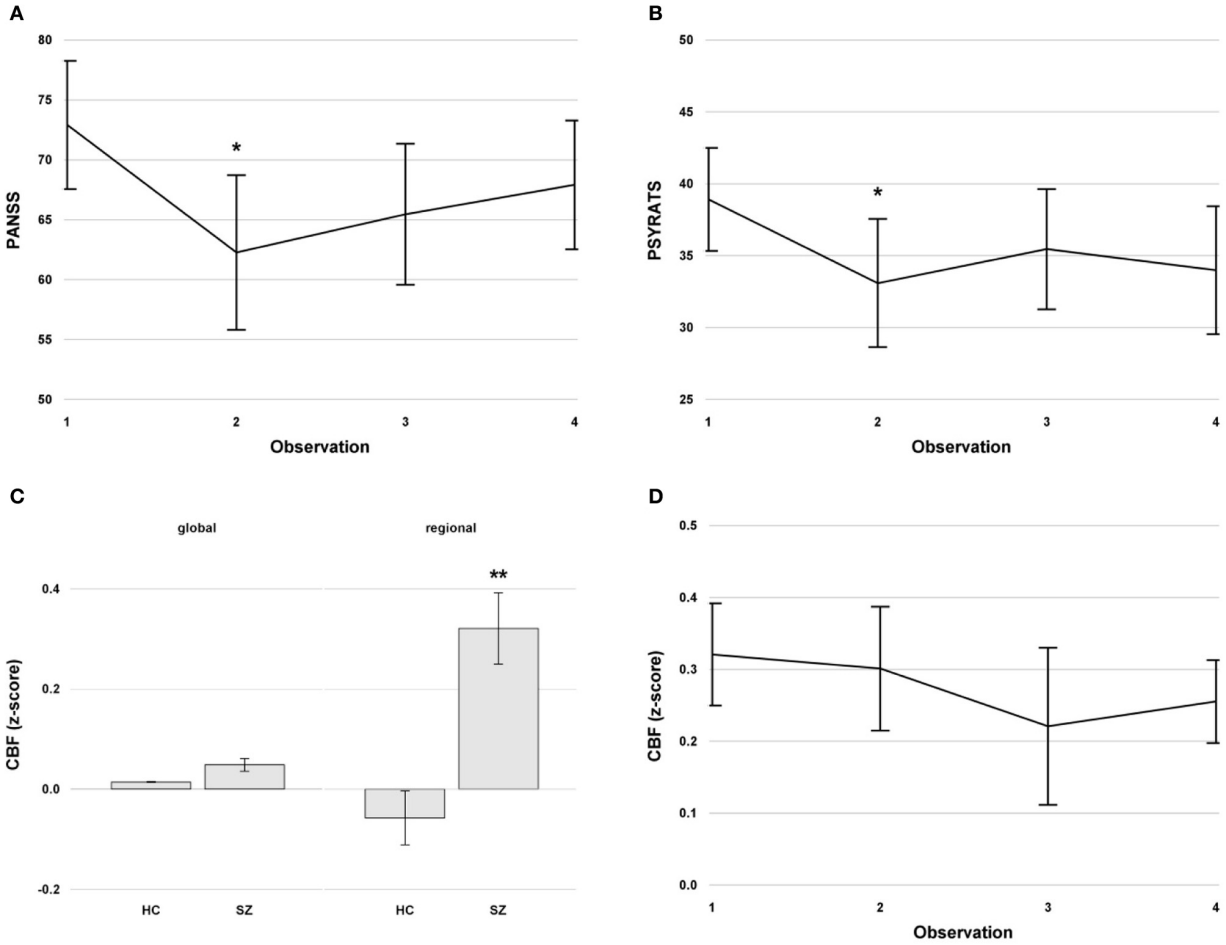
**(A)** Mean PANSS scores with standard errors across time in the patients. **(B)** Mean PSYRATS scores with standard errors across time in the patients. **(C)** Mean global and regional cerebral blood flow (CBF) in the left superior temporal gyrus with standard error at baseline in healthy controls and patients with schizophrenia with persistent auditory verbal hallucinations. **(D)** Mean regional CBF with standard errors in the left superior temporal gyrus across time in the patients. The CBF values were z-transformed. ^**^indicates a significant difference at *p* < 0.01, ^*^indicates a significant difference at *p* < 0.05. Abbreviations: PANSS, Positive and Negative Syndrome Scale; PSYRATS, Psychotic Symptoms Rating Scale; CBF, cerebral blood flow. HC, Healthy Controls; SZ, Schizophrenia.

Eleven patients and 19 healthy controls were measured at baseline. Three additional repeated measurements were conducted on the patients. The intervals between measurements differed across the patient group (mean ± SD, 28.2 ± 32.9 days). Altogether, 44 measurements were conducted in the patients. The clinical and demographic characteristics of the subject sample are detailed in Table 1. The mean CPZE at baseline was 714.5 ± 475.5 mg and remained stable during the study. Between the first and second measurement, which was after the TMS treatment, the PANSS [*F*_(1, 10)_ = 14.88, *p* = 0.003; Figure 2A] and PSYRATS [*F*_(1, 10)_ = 9.97, *p* = 0.01; Figure 2B] scores were decreased significantly in the patients. No effect of TMS stimulation mode (1 Hz vs. theta burst) was found on the PANSS [*F*_(1, 10)_ = 0.18, *p* = 0.7] and PSYRATS [*F*_(1, 10)_ = 1.19, *p* = 0.3] scores, and no effect of medication was evident. The PSYRATS score remained stable after TMS treatment across all further measurements, which included observations at *t* = 2, 3, and 4 [*F*_(2, 19)_ = 1.04, *p* = 0.37; Figure 2B]. The PANSS scores displayed a trend toward a time effect after TMS, which included observations at *t* = 2, 3, and 4, [*F*_(2, 19)_ = 3.18, *p* = 0.06; Figure 2A].

**Table 1:** **Subject characteristics of the patients (*n* = 11) and healthy controls (*n* = 19)**.

**Characteristic**	**Patients (*n* = 11)**	**Healthy controls (*n* = 19)**	**Test statistic**	***p*-value**
Sex, F/M	8/3	11/8	Fisher's exact	0.5
Diagnosis	11 Sz	n.a.	n.a.	n.a.
Age, mean (SD), y	37.1 (8.8)	38.5 (12.2)	t-test	0.7
Age at onset, mean (SD), y	23.3 (4.4)	n.a.	n.a.	n.a.
Chlorpromazine equivalent dose at study entry, mean (SD)	714.5 (475.5)	n.a.	n.a.	n.a.
Global mean cerebral blood flow corrected for gray matter at study entry, mean (SD)	65.7 (7.7)	67.1 (6.2)	*t*-test	0.6
PANSS score at study entry, mean (SD)	67.1 (18.9)	n.a.	n.a.	n.a.
PSYRATS score at study entry, mean (SD)	35.4 (2.0)	n.a.	n.a.	n.a.

Values are presented as means ± SD. Abbreviations: F, female; M, male; Sz, schizophrenia (according to ICD-10); n.a., not available; PANSS, Positive and Negative Syndrome Scale; SD, standard deviation; PSYRATS, Psychotic Symptoms Rating Scale; CBF, cerebral blood flow.

### Global and regional CBF at baseline

The pooled CBF (global and regional) was significantly higher in patients with schizophrenia compared to healthy controls [*F*_(1, 28)_ = 21.19, *p* < 0.0001], and the regional CBF in the left STG was significantly higher in the entire sample [*F*_(1, 28)_ = 5.02, *p* < 0.04]. The localization-by-diagnosis interaction was also significant [*F*_(1, 28)_ = 14.74, *p* < 0.0007]. The *post-hoc* tests revealed a significantly higher regional CBF in patients compared to controls [*t*_(1, 28)_ = 5.97, *p* < 0.0001, Figure 2C]. Furthermore, regional CBF in the left STG in patients was significantly higher compared to global CBF [*t*_(1, 28)_ = 3.82, *p* < 0.004, Figure 2C], an effect that was not found in healthy controls [*t*_(1, 28)_ = 1.32, *p* = 0.6].

### Longitudinal changes of global and regional CBF

The regional CBF in the left STG was significantly higher compared to the global CBF in patients across time [*F*_(1, 10)_ = 24.67, *p* < 0.0007]. There was no time effect [*F*_(3, 30)_ = 0.98, *p* = 0.4], no effect of interval [*F*_(1, 69)_ = 0.01, *p* = 0.9], and no time-by-localization interaction [*F*_(3, 30)_ = 0.23, *p* = 0.9] in the longitudinal patient data (Figure 2D). In addition, no TMS effect was evident in the left STG CBF for the first 2 measurements [*F*_(1, 10)_ = 0.1, *p* = 0.75]. After TMS with *t* = 2, 3, and 4, CBF in the left STG of patients was still increased compared to the baseline CBF of healthy controls [at *t* = 2: *t*_(1, 28)_ = 5.27, *p* < 0.0001; at *t* = 3: *t*_(1, 28)_ = 3.62, *p* = 0.001; at *t* = 4: *t*_(1, 28)_ = 5.3, *p* < 0.0001]. Furthermore, no medication effect of CPZE was evident [*F*_(1, 9)_ = 0.15, *p* = 0.7].

### Correlation of regional CBF and psychopathology

The regional CBF in the left STG was negatively associated with the PSYRATS scores across time [*F*_(1, 28)_ = 9.93, *p* = 0.004]. No such association was found between CBF and the PANSS scores [*F*_(1, 28)_ = 1.7, *p* = 0.2].

## Discussion

Until now, longitudinal studies of global and regional CBF in patients suffering from schizophrenia and AVH have not been conducted. In this study, we were able to investigate patients who were suffering from AVH several times during the course of their disease, and we were able to demonstrate that the patients had significantly higher CBF in a predefined region, the left STG, compared to healthy controls and compared to global CBF. Furthermore, the increase in regional CBF was a stable feature across time that was unaffected by treatment with TMS.

The aim of the current study was to gain further insight into the involvement of the left STG in AVH. Indeed, this region is thought to play a key role in AVH interventions. Previous studies have suggested that the left STG might be an appropriate target region in TMS in patients with schizophrenia and AVH. Ten days of TMS treatment to the left STG resulted in a clinically relevant improvement of symptoms as measured by the PSYRATS, and this has been shown to be correlated with decreases in the CBF in primary language and auditory regions (Kindler et al., 2013) but not with CBF changes in the STG. Furthermore, responders and non-responders to TMS were identified by the CBF in the left STG before treatment. One aim of the current study therefore was to search for fluctuations in the regional CBF in the left STG that would be consistent with the proposed responsiveness to TMS (Homan et al., 2012). However, we did not find fluctuations in the regional CBF but a persistent hyperperfusion in the left STG during all 4 measurements compared to the baseline CBF of healthy controls and the global CBF, suggesting that increased regional CBF in the left STG is a trait and not a state marker in patients with schizophrenia and AVH. This was further supported by the fact that treatment with TMS between the first and second measurements did not alter CBF in the left STG, which was in contrast to previous findings of decreased regional glucose metabolism at the stimulation site in the left STG after 10 days of TMS (Horacek et al., 2007). However, the finding of the latter study was located more anteriorly and more inferiorly (at the temporal pole) than the ROI in the current study. Thus, the current finding might indicate that patients who have regional CBF values below the previously proposed cutoff value (Homan et al., 2012) are unlikely to change to a responsive state across time.

In addition, the findings of continuously increased left STG CBF in patients with AVH provided further evidence of the involvement of the left STG in AVH, which has also been shown for patients with AVH of epileptic etiology (Hauf et al., 2013). It has been suggested that AVH arise from a disorder of inner verbal experiences, particularly from a disorder of the monitoring of inner speech (David, 1994; McGuire et al., 1995). A bottom–up model has been proposed that includes alterations in secondary and sometimes even primary sensory cortices, speech production and reception areas (inferior frontal gyrus, left STG) and in the coupling with monitoring areas (anterior cingulate) (Allen et al., 2008). Specifically, alterations in the connectivity of the STG, the inferior frontal gyrus, and the anterior cingulate cortex might be the precondition for altered activity in language processing areas (Allen et al., 2008). Thus, in the aforementioned model, AVH are thought to be associated with decreased top–down control by the ventral anterior cingulate, prefrontal, premotor, and cerebellar cortices and failure in monitoring and volitional assignment. Together, these alterations might be the neuronal basis of the experience of perceptions without sensory stimuli (Allen et al., 2008). This is in line with previous research that has focused on the possible therapies of AVH. With regard to therapy, it is known that low-frequency repetitive TMS that is delivered to the left temporoparietal cortex, which is a brain area that is critical to speech perception, reduces AVH (Hoffman et al., 2000, 2003; Aleman et al., 2007). Recently, the first case report was published of a patient with schizophrenia and medication-resistant AVH who was successfully stimulated at the same region with transcranial direct current stimulation (Homan et al., 2011), and this finding has been confirmed by a larger study that used a slightly different stimulation paradigm (Brunelin et al., 2012).

The present study conducted repeated measurements of psychopathology and CBF. Our finding of persistent regional hyperperfusion in the left STG in AVH was consistent with a recent meta-analysis that has suggested that neuronal activity in the left STG is a trait marker in AVH (Kuhn and Gallinat, 2012). That study classified neuroimaging studies that explored AVH in state and trait studies. Symptom-catching state studies compare periods of the presence of hallucinations with periods of the absence of hallucinations within subject, whereas trait studies compare the brain activity between hallucinating subjects with that of non-hallucinating patients with schizophrenia or healthy controls. The findings in the meta-analysis have supported the idea that AVH are caused by a permanent defective monitoring of inner speech (trait) and a temporary misattribution of internally generated speech (state). State-like brain regions have been consistently found in frontal speech-generating regions, whereas the left temporal brain regions (left STG, left middle temporal gyrus) have been found in trait studies. The alteration that was found in these left temporal regions was less activation during tasks involving verbal material-like inner speech generation or prerecorded listening. However, the direction of this alteration has been somewhat difficult to interpret because brain activity is first compared on a within-subject basis, and this difference is then contrasted between participants. This raised the question of whether hallucinating patients could have tonically high activity or tonically low activity that is independent of state (the degree of subjectively experienced AVH) in the temporal lobe, which would then contribute to the small differences that were observed in the within-subject comparison between conditions and to the seemingly decreased activation in hallucinating subjects compared to non-hallucinating subjects (Kuhn and Gallinat, 2012). Thus, our findings of persistently increased CBF in the left STG was in line with the suggestion of tonically high activity in hallucinating patients and extends the proposed trait marker to the confirmation of the increased hyperperfusion, at least for our 4 measurement time points.

Some limitations of our study design merit comment. No direct relationship between brain activity and the occurrence of hallucinations during scanning was investigated because, in ASL, the mean cerebral perfusion was calculated over the scanning time. Therefore, the temporal resolution was low. In addition, the variance of the intervals between the measurements was certainly a weakness of the present study. However, we took this variance into account by including the intervals between measurements as a fixed effect in the general linear mixed models that we computed, and no effect was evident. In addition, ASL measured a baseline CBF increase, which can be interpreted as a trait for AVH. However, because we did not exactly know when during the measurements that the patients were hallucinating, one cannot exclude that this region also can show state-dependent behavior like that previously described for the primary auditory cortex (Dierks et al., 1999; Jardri et al., 2011; Kompus et al., 2011). As long as a ceiling for neural activity for a certain region has not been reached, a region with a higher baseline CBF can generate additional neuronal activation. Finally, the small sample size has to be considered. The study might thus have been underpowered for detecting significant differences in the left STG CBF across time.

In conclusion, this study showed that the regional CBF in the left STG was tonically increased in patients with schizophrenia and AVH, and this was consistent with what has previously been termed a trait marker of AVH in schizophrenia.

### Conflict of interest statement

The authors declare that the research was conducted in the absence of any commercial or financial relationships that could be construed as a potential conflict of interest.
